# CVtreeMLE: Efficient Estimation of Mixed Exposures using Data Adaptive Decision Trees and Cross-Validated Targeted Maximum Likelihood Estimation in R

**DOI:** 10.21105/joss.04181

**Published:** 2023-02-21

**Authors:** David McCoy, Alan Hubbard, Mark Van der Laan

**Affiliations:** 1 Division of Environmental Health Sciences, University of California, Berkeley, CA, United States of America; 2 Department of Biostatistics, University of California, Berkeley, CA, United States of America

## Abstract

Statistical causal inference of mixed exposures has been limited by reliance on parametric models and, until recently, by researchers considering only one exposure at a time, usually estimated as a beta coefficient in a generalized linear regression model (GLM). This independent assessment of exposures poorly estimates the joint impact of a collection of the same exposures in a realistic exposure setting. Marginal methods for mixture variable selection such as ridge/lasso regression are biased by linear assumptions and the interactions modeled are chosen by the user. Clustering methods such as principal component regression lose both interpretability and valid inference. Newer mixture methods such as quantile g-computation (Keil et al., 2020) are biased by linear/additive assumptions. More flexible methods such as Bayesian kernel machine regression (BKMR)(Bobb et al., 2014) are sensitive to the choice of tuning parameters, are computationally taxing and lack an interpretable and robust summary statistic of dose-response relationships. No methods currently exist which finds the best flexible model to adjust for covariates while applying a non-parametric model that targets for interactions in a mixture and delivers valid inference for a target parameter. Non-parametric methods such as decision trees are a useful tool to evaluate combined exposures by finding partitions in the joint-exposure (mixture) space that best explain the variance in an outcome. However, current methods using decision trees to assess statistical inference for interactions are biased and are prone to overfitting by using the full data to both identify nodes in the tree and make statistical inference given these nodes. Other methods have used an independent test set to derive inference which does not use the full data. The CVtreeMLE R package provides researchers in (bio)statistics, epidemiology, and environmental health sciences with access to state-of-the-art statistical methodology for evaluating the causal effects of a data-adaptively determined mixed exposure using decision trees. Our target audience are those analysts who would normally use a potentially biased GLM based model for a mixed exposure. Instead, we hope to provide users with a non-parametric statistical machine where users simply specify the exposures, covariates and outcome, CVtreeMLE then determines if a best fitting decision tree exists and delivers interpretable results.

Although users do not need strong knowledge of the underlying theory, CVtreeMLE builds off the general theorem of cross-validated minimum loss-based estimation (CV-TMLE) which allows for the full utilization of loss-based ensemble machine learning to obtain the initial estimators needed for our target parameter without risk of overfitting. CVtreeMLE uses V-fold cross-validation and partitions the full data into parameter-generating samples and estimation samples. For example, when V=10, integers 1–10 are randomly assigned to each observation with equal probability. In fold 1, observations assigned to 1 are used in the estimation sample and all other observations are used in the parameter-generating sample. This process rotates through the data until all the folds are complete. In the parameter-generating sample, decision trees are applied to a mixed exposure to obtain rules and estimators are created for our statistical target parameter. The rules from decision trees are then applied to the estimation sample where the statistical target parameter is estimated. CVtreeMLE makes possible the non-parametric estimation of the causal effects of a mixed exposure producing results that are both interpretable and guaranteed to converge to the truth (under assumptions) at a particular rate as sample size increases. Additionally, CVtreeMLE allows for discovery of important mixtures of exposure *and also* provides robust statistical inference for the impact of these mixtures.

## Statement of Need

In many disciplines there is a demonstrable need to ascertain the causal effects of a mixed exposure. Advancement in the area of mixed exposures is challenged by real-world joint exposure scenarios where complex agonistic or antagonistic relationships between mixture components can occur. More flexible methods which can fit these interactions may be less biased, but results are typically difficult to interpret, which has led researchers to favor more biased methods based on GLM’s. Current software tools for mixtures rarely report performance tests using data that reflect the complexities of real-world exposures (Carlin et al., 2013; Keil et al., 2020; Yu et al., 2022). In many instances, new methods are not tested against a ground-truth target parameter under various mixture conditions. New areas of statistical research, rooted in non/semi-parametric efficiency theory for statistical functionals, allow for robust estimation of data-adaptive parameters. That is, it is possible to use the data to both define and estimate a target parameter. This is important in mixtures when the most important set of variables and levels in these variables are almost always unknown. Thus, the development of asymptotically linear estimators for data-adaptive parameters are critical for the field of mixed exposure statistics. However, the development of open-source software which translates semi-parametric statistical theory into well-documented functional software is a formidable challenge. Such implementation requires understanding of causal inference, semi-parametric statistical theory, machine learning, and the intersection of these disciplines. The CVtreeMLE R package provides researchers with an open-source tool for evaluating the causal effects of a mixed exposure by treating decision trees as a data-adaptive target parameter to define exposure. The CVtreeMLE package is well documented and includes a vignette detailing semi-parametric theory for data-adaptive parameters, examples of output, results with interpretations under various real-life mixture scenarios, and comparison to existing methods.

## Background

In many research scenarios, the analyst is interested in causal inference for an *a priori* specified treatment or exposure. This is because when a single exposure/treatment is measured the analyst is interested in understanding how this exposure/treatment impacts an outcome, controlling for covariates. However, in the evaluation of a mixed exposure, such as air pollution or pesticides, it is not possible to estimate the expected outcome given every combination of exposures. This is because the conditional outcome given every combination of exposures is not measured. Furthermore, it is likely that, only certain exposures within a mixture have marginal or interacting effects on an outcome. In such a setting, new methods are needed for statistical learning from data that go beyond the usual requirement that the estimand is *a priori* defined in order to allow for proper statistical inference (Hubbard et al., 2016). In the case of mixtures, it is necessary to map a set of continuous mixture components into a lower dimensional representation of exposure using a pre-determined algorithm, and then estimate a target parameter on this more interpretable exposure. Decision trees provide a useful solution by mapping a set of exposures into a rule which can be represented as a binary vector. This binary vector indicates whether an individual has been exposed to a particular rule estimated by the decision tree. Our target parameter is then defined as the mean difference in counterfactual outcomes for those exposed to the mixture subspace (delineated by the rule) compared to those unexposed, or the average treatment effect (ATE) for the mixed exposure. Decision trees have been used as a data-adaptive parameter to explore and estimate heterogeneous treatment effects of a binary treatment (Athey & Imbens, 2016). Using a so-called “honest” approach, this method estimates the treatment effect in subpopulations based on covariates in a left-out sample. This approach is limited by not making use of the full data and not data-adaptively selecting the best decision tree. Advancements in using decision trees as a data-adaptive parameter that solve both these issues and guarantees nominal confidence interval coverage under certain assumptions are needed. Under normal assumptions of conditional independence (A is independent of Y given W) and positivity (enough experimentation in the data) identifiability of the ATE causal parameter is obtained from the observed data via the statistical functional for a data adaptively determined exposure. This is because, 1. by using Super Learner as our estimator, we are asymptotically guaranteed to select the correct functional for the underlying joint distribution thereby removing bias due to model error and 2. by using TMLE we debias our initial counterfactual for the ATE to target the parameter of interest. The remaining potential bias is therefore due to aggregated data and not the statistical method.

### CVtreeMLE’s Scope

Building on prior work related to data-adaptive parameters (Hubbard et al., 2016) and CV-TMLE (van der Laan & Rose, 2011), chapter 27. CVtreeMLE is a novel approach for estimating the joint impact of a mixed exposure by using cross-validated targeted minimum loss-based estimation which guarantees consistency, efficiency, and multiple robustness despite using highly flexible learners to estimate a data-adaptive parameter. CVtreeMLE summarizes the effect of a joint exposure on the outcome of interest by first doing an iterative backfitting procedure, similar to generalized additive models, to fit f(A), a Super Learner of decision trees, and h(W), an unrestricted Super Learner, in a semi-parametric model; E(Y∣A,W)=f(A)+h(W), where A is a vector of exposures and W is a vector of covariates. In this way, we can data-adaptively find the best fitting decision tree model which has the lowest cross-validated model error while flexibly adjusting for covariates. This procedure is done to find rules for the mixture modeled collectively and for each mixture component individually. There are two types of results, 1. an ATE comparing those who fall within a subspace of the joint exposure versus those in the complement of that space and 2. the ATE for each data-adaptively identified threshold of an individual mixture component when compared to the lowest identified exposure level. The CVtreeMLE software package, for R (R Core Team, 2020), implements this methodology for deriving causal inference from ensemble decision trees.

CVtreeMLE is designed to provide analysts with both V-fold specific and pooled results for ATE causal effects of a joint exposure determined by decision trees. It integrates with the sl3 package (Coyle et al., 2020) to allow for ensemble machine learning to be leveraged in the estimation of nuisance parameters.

### Availability

The CVtreeMLE package has been made publicly available via GitHub. Use of the CVtreeMLE package has been extensively documented in the package’s README and a vignette.
